# Interaction Effect between Phosphorus and Zinc on their Availability in Soil in Relation to their Contents in Stevia (*Stevia rebaudiana*)

**DOI:** 10.1100/tsw.2005.63

**Published:** 2005-06-26

**Authors:** Kuntal Das, Raman Dang, T. N. Shivananda, Pintu Sur

**Affiliations:** ^1^ Al-Ameen College of Pharmacy, Hosur Road, Bangalore-560027, Karnataka, India; ^2^ Indian Institute of Horticultural Research, Hessaraghatta, Bangalore, Karnataka, India; ^3^ Department of Agricultural Chemistry and Soil Science, Bidhan Chandra Krishi Viswavidyalaya, Mohanpur–741252, Nadia, West Bengal, India

**Keywords:** interaction, phosphorus, soil, stevia, zinc

## Abstract

A greenhouse experiment was conducted at the Indian Institute of Horticultural Research (IIHR), Bangalore to study the interaction effect between phosphorus and zinc on their availability in soil in relation to their contents in stevia (*Stevia rebaudiana*). The results show that the amount of available P and Zn content in soil has been found to increase initially and, thereafter, the amount of the same decreased with the progress of plant growth up to 60 days irrespective of treatments. The amount of P and Zn in soils showed an increase with their separate applications either as soil or foliar spray while that of the same value significantly decreased both in soils and plants due to their combined applications, suggesting a mutual antagonistic effect between Zn and P affecting each other's availability in soil and content in the stevia plant.

## INTRODUCTION

Stevia (*Stevia rebaudiana* Bertoni) is a sweet herb (medicinal plant) native of Paraguay. It belongs to the family Compositae and is fast becoming a major source of high-potency biosweetener for the rapidly growing market of "natural" foods, replacing chemical sweeteners (Saccharine) and even table sugar[1]. Roy et al.[2] also reported that the leaves of stevia are free from carbohydrates and calories and, hence, it can be used safely by diabetic patients. It has now been recognized that stevia has many uses for human beings, especially when it acts as a sugar substitute for those persons with blood sugar problems.

In view of the above fact, cultivation of stevia is gradually gaining importance in India, having no sufficient agronomic management practices. Any practice of a nutrient management, which either decreases or increases the supply of another nutrient element or its absorption from the soil by plants or translocation and mobility within the plant, will influence its nutrition and, thereby, the nutrient use efficiency and crop yields[3]. The use of NPK fertilizers and well-rotten FYM are common practice for cultivation of any crop including medicinal plants like stevia. However, the efficiency of applied P rarely exceeds 30% and that of most of the micronutrient cations more than 10%. Therefore, their repeated applications over the years lead to their buildup and interactions in soils and plants, affecting agricultural production[3]. But the use of micronutrient fertilizers, especially Zn, for cultivation is still limited; rather, no information about this is available. The heavy use of P fertilizers may have some adverse or favourable effect on the availability of applied Zn in soils as well as its effect on plants[3,4]. Hence, the interaction effect between Zn and P is still very much contradictory. Therefore, it is worthwhile to study the interaction effect between P and Zn on their availability in soil in relation to their contents in stevia.

## MATERIALS AND METHODS

Cuttings of the stevia plant were collected from Gandhi Krishi Vigyan Kendra, Bangalore and were used as a test plant. Before planting, initial soil samples were analysed for pH (Soil:Water, 1:2.5), organic carbon, CEC, available P, and DTPA-Zn by following the methods described by Jackson[5] and Lindsay and Norvell[6]. After extracting the soil samples, Zn and P were determined with the help of an atomic absorption spectrophotometer (Perkin Elmer model AAnalyst 100) and spectrophotometer, respectively. The relevant physicochemical properties of soils were: pH, 8.9; organic carbon, 3.8 g/kg; available $P_2O_5$, 38 kg/ha; CEC, 14.4 Cmol($p^+$)/kg; DTPA-extractable Zn, 0.42 mg/kg.

Thirty-two earthen pots (15-kg capacity) were taken and 10-kg powdered soil collected from the Indian Institute of Horticultural Research Farm, Hessaraghata, Bangalore was filled in each pot and the following treatments were: $T_1$ – absolute control, no application of Zn and P; $T_2$ – application of $P_2O_5$, but no application of Zn; $T_3$ – soil application of Zn as $ZnSO_4$ @ 10 kg/ha, but no application of $P_2O_5$; $T_4$ – foliar application of Zn as $ZnSO_4$ @ 0.2% solution, but no application of $P_2O_5$; $T_5$ – both soil ($ZnSO_4$ @ 10 kg/ha) and foliar ($ZnSO_4$ @ 0.2%) of Zn, but no application of P; $T_6$ – soil application of both Zn as $ZnSO_4$ @ 10 kg/ha and $P_2O_5$ as single super phosphate (SSP) @ 30 kg/ha; $T_7$ – foliar application of Zn as $ZnSO_4$ @ 0.2% along with soil application of $P_2O_5$ at 30 kg/ha; $T_8$ – both soil (Zn as $ZnSO_4$ @ 10 kg/ha) and foliar (Zn as $ZnSO_4$ @ 0.2%) application of Zn along with basal application of $P_2O_5$ as SSP @ 30 kg/ha. Each treatment was replicated four times in a completely randomised design (CRD). There were 32 pots (8 × 4) altogether. The pots were placed in net house in order to monitor growth of the plant after cuttings of the stevia plant were put in each pot. Then the plants were allowed to grow for a period of 60 days. The periodic collection of soil and plant samples was made and analysed for pH, DTPA-extractable Zn, and available P by following the method as mentioned earlier.

## RESULTS AND DISCUSSION

The results (Table 1) show that the amount of available P content in soil has been found to increase initially and, thereafter, the amount of the same gradually decreased up to 60 days of plant growth irrespective of treatments. The magnitude of such changes, however, varied with treatments; the greater amount in the treatment where only P was applied as basal. The results further indicated that the increased amount of available P was found to be significantly decreased with the application of Zn and P; the greater decrease (1.74 mg/kg) with both soil and foliar application of Zn along with basal application of P to the soil at 60 days of plant growth. Such decrease might be due to the antagonistic effect between Zn and P in soils forming insoluble compounds, $Zn_3(PO_4)_2$ resulting in the low amount of P in the available pool[3,7,8].

**TABLE 1**
**Interaction Effect Between P and Zn on the Changes in Available P Content (mg/kg) in Soil**

| Treatments | Days after Plant Growth | | | | |
|---|---|---|---|---|---|
| | 15 | 30 | 45 | 60 | Mean |
| –Zn, –P ($T_1$) | 1.64 | 2.14 | 1.86 | 1.74 | 1.85 |
| –Zn, +P ($T_2$) | 3.26 | 3.68 | 2.48 | 1.97 | 2.85 |
| Zn(S), –P ($T_3$) | 1.58 | 1.79 | 1.57 | 1.46 | 1.6 |
| Zn(F), –P ($T_4$) | 1.49 | 1.55 | 1.50 | 1.44 | 1.50 |
| Zn(S+F), –P ($T_5$) | 1.60 | 1.64 | 1.54 | 1.48 | 1.57 |
| Zn(S), +P ($T_6$) | 2.78 | 2.96 | 2.32 | 1.84 | 2.48 |
| Zn(F), +P ($T_7$) | 2.66 | 2.88 | 2.41 | 1.81 | 2.44 |
| Zn(S+F), +P ($T_8$) | 2.69 | 2.77 | 2.11 | 1.74 | 2.33 |
| Mean | 2.21 | 2.43 | 1.97 | 1.69 | |
| *p* (0.05) | 0.14 | 0.21 | 0.17 | 0.11 | |

The results (Table 2) show that the amount of DTPA-extractable Zn content also showed almost a similar trend of changes to that of available P, but the absolute amount of Zn was recorded much lower than that of available P in soil. Considering the effect of different treatments, it was found that the amount of Zn content in soil was maintained highest (1.36 mg/kg) in the treatment where Zn was applied as both soil and foliar spray in the absence of P, which was closely followed by the treatment $T_3$ (1.22 mg/kg) where Zn was applied to the soil in the absence of P. The relatively lower amount of Zn was maintained in treatments where combined application of Zn, both as soil and foliar, and P was made. Such decrease in the amount of Zn due to combined application of P and Zn might be explained as the antagonistic effect between them. It has been reported that the interaction between Zn and P occurred in soil because added P decreased the available Zn content in plants. An increasing level of P in soils significantly decreased shoots and grain yields of maize[3].

**TABLE 2**
**Interaction Effect Between P and Zn on the Changes in Zn Content (mg/kg) in Soil**

| Treatments | Days after Plant Growth | | | | |
|---|---|---|---|---|---|
| | 15 | 30 | 45 | 60 | Mean |
| –Zn, –P ($T_1$) | 0.44 | 0.52 | 0.43 | 0.46 | 0.46 |
| –Zn, +P ($T_2$) | 0.46 | 0.50 | 0.44 | 0.48 | 0.47 |
| Zn(S), –P ($T_3$) | 0.82 | 1.47 | 1.78 | 1.22 | 1.32 |
| Zn(F), –P ($T_4$) | 0.48 | 0.55 | 0.68 | 0.62 | 0.58 |
| Zn(S+F), –P ($T_5$) | 0.88 | 1.54 | 1.89 | 1.36 | 1.42 |
| Zn(S), +P ($T_6$) | 0.70 | 0.82 | 0.86 | 0.88 | 0.81 |
| Zn(F), +P ($T_7$) | 0.54 | 0.63 | 0.59 | 0.51 | 0.57 |
| Zn(S+F), +P ($T_8$) | 0.79 | 0.82 | 0.89 | 0.83 | 0.83 |
| Mean | 0.63 | 0.86 | 0.95 | 0.80 | |
| *p* (0.05) | 0.16 | 0.12 | 0.28 | 0.23 | |

The results (Table 3) show that the amount of Zn in stevia plants gradually decreases after attaining a maximum value at 30 days of plant growth, which might be due to the dilution effect resulting from the greater biomass production at the later period of growth. The amount of Zn content was maintained highest (4.62 mg/kg) in the treatment $T_5$ where Zn was applied as both soil and foliar spray in the absence of P with the simultaneous highest total biomass production (23.34 g), which suggests that the contribution of Zn content in plants towards biomass production was far greater than that of P content. However, such decrease in Zn content in stevia plants due to P application might be explained by restricting the translocation of Zn from roots to other parts of the stevia plant resulting from the interference of applied P in the metabolic processes of plants[3]. An excess of P inhibits Zn uptake, first by curtailing its translocation through endodermis into root xylem, and finally and more importantly, by lowering its rate of absorption through the epidermal or surface cell layer of the root[9].

**TABLE 3**
**Interaction Effect Between P and Zn on the Zn Content (mg/kg) in Plants**

| Treatments | Days after Plant Growth | | | | |
|---|---|---|---|---|---|
| | 15 | 30 | 45 | 60 | Mean |
| –Zn, –P ($T_1$) | 2.61 | 2.82 | 2.58 | 2.10 | 2.53 |
| –Zn, +P ($T_2$) | 2.30 | 2.48 | 2.27 | 1.96 | 2.25 |
| Zn(S), –P ($T_3$) | 4.54 | 4.86 | 4.42 | 4.28 | 4.5 |
| Zn(F), –P ($T_4$) | 4.91 | 5.18 | 4.76 | 4.40 | 4.81 |
| Zn(S+F), –P ($T_5$) | 4.98 | 5.89 | 4.78 | 4.62 | 5.07 |
| Zn(S), +P ($T_6$) | 2.58 | 2.78 | 2.36 | 2.11 | 2.46 |
| Zn(F), +P ($T_7$) | 4.23 | 4.58 | 3.82 | 3.77 | 4.1 |
| Zn(S+F), +P ($T_8$) | 2.48 | 2.86 | 2.24 | 2.08 | 2.42 |
| Mean | 3.58 | 3.93 | 3.40 | 3.17 | |
| *p* (0.05) | 1.12 | 1.26 | 1.14 | 1.22 | |

The results (Table 4) show that the amount of P content in stevia plants has been found to increase initially and, thereafter, the amount of the same decreased up to 60 days of plant growth. Such variation in the P content, however, varied with treatments. The highest (1.98 mg/kg) and lowest (1.46 mg/kg) amount of P was maintained in $T_7$ and $T_4$ treatments where Zn was applied as foliar along with basal application of P and foliar spray of Zn in the absence of basal application P, respectively. Zinc fertilization depressed P concentration in plants, but the interactive effect of P on Zn was more pronounced than that of Zn on P[3]. It was also reported that reduced synthesis of some organic acid complexes at high P levels partly might be the cause for P-induced Zn deficiency and also partly due to reduction of unit absorption rate of Zn by roots putting restraint on the functional requirement of Zn by plants[9].

## CONCLUSIONS

The interaction effect between P and Zn did not show any positive effect on their availability in soil as well as their contents in the stevia (*S. rebaudiana*) plant. The results clearly suggested that the application of Zn only, as both soil and foliar spray, was found superior over that of P only as basal application in relation to their availability as well as contents in plants, while that of the same content in soil and plant was recorded significantly lowest when Zn was applied as both soil and foliar spray in the presence of P, suggesting an antagonistic relationship between Zn and P in relation to their availability in soil and contents in plants.

**TABLE 4**
**Interaction Effect Between P and Zn on the P Content (mg/kg) in Plant**

| Treatments | Days after Plant Growth | | | | |
|---|---|---|---|---|---|
| | 15 | 30 | 45 | 60 | Mean |
| –Zn, –P ($T_1$) | 1.74 | 1.92 | 1.80 | 1.58 | 1.76 |
| –Zn, +P ($T_2$) | 2.40 | 2.68 | 2.42 | 1.94 | 2.36 |
| Zn(S), –P ($T_3$) | 1.76 | 1.89 | 1.77 | 1.60 | 1.76 |
| Zn(F), –P ($T_4$) | 1.78 | 1.86 | 1.80 | 1.46 | 1.73 |
| Zn(S+F), –P ($T_5$) | 1.71 | 1.88 | 1.78 | 1.58 | 1.74 |
| Zn(S), +P ($T_6$) | 1.96 | 2.05 | 1.84 | 1.71 | 1.89 |
| Zn(F), +P ($T_7$) | 2.37 | 2.74 | 2.48 | 1.98 | 2.39 |
| Zn(S+F), +P ($T_8$) | 1.97 | 2.10 | 1.87 | 1.73 | 1.92 |
| Mean | 1.96 | 2.14 | 1.97 | 1.70 | |
| $p$ (0.05) | 0.38 | 0.27 | 0.18 | 0.21 | |

## ACKNOWLEDGEMENTS

The authors are grateful to the Department of Agricultural Chemistry and Soil Science, Bidhan Chandra Krishi Viswavidyalaya, Mohanpur, Nadia, West Bengal for rendering help and cooperation in analysing soil and plant samples with the help of an atomic absorption spectrophotometer (Perkin Elmer Model AAnalyst 100) during the course of the present investigation as a part of postgraduate dissertation work.

## BIOSKETCHES

**Kuntal Das**, lecturer in pharmacognosy in St. John's Pharmacy College, Bangalore – 40, India. Sri Das has published seven research papers relating to medicinal and aromatic plants with particular reference to stevia.

**Raman Dang** is an assistant professor in the Department of Pharmacognosy, Al-Ameen College of Pharmacy, Hosur Road, Bangalore – 560027, Karnataka, India. Dr. Dang has published some good-quality research papers on medicinal and aromatic plants with special reference to pharmaceutical research.

**T.N. Shivananda** is a senior scientist in the Division of Medicinal and Aromatic Plants, Indian Institute of Horticultural Research, Hessaraghatta, Bangalore, Karnataka, India. Dr. Shivananda has published a good number of original research papers in journals of national and international repute.

**Pintu Sur** is a Ph.D. student in the Department of Agricultural Chemistry and Soil Science, Bidhan Chandra Krishi Viswavidyalaya, Mohanpur – 741252, Nadia, West Bengal, India whose field of specialization is soil chemistry, soil fertility, and plant nutrition. Sri Sur has published three research papers relating to soil chemistry and plant nutrition with particular reference to micronutrients.
